# An enhanced pattern detection and segmentation of brain tumors in MRI images using deep learning technique

**DOI:** 10.3389/fncom.2024.1418280

**Published:** 2024-06-26

**Authors:** Lubna Kiran, Asim Zeb, Qazi Nida Ur Rehman, Taj Rahman, Muhammad Shehzad Khan, Shafiq Ahmad, Muhammad Irfan, Muhammad Naeem, Shamsul Huda, Haitham Mahmoud

**Affiliations:** ^1^Qurtuba University of Science and Information Technology, Peshawar, Pakistan; ^2^Abbottabad University of Science and Technology, Abbottabad, Pakistan; ^3^Institute of Management Sciences (IMSciences), Peshawar, Pakistan; ^4^Department of Industrial Engineering, College of Engineering, King Saud University, Riyadh, Saudi Arabia; ^5^Department of Computer Science, Kohat University of Science and Technology, Kohat, Pakistan; ^6^School of Information Technology, Deakin University, Burwood, VIC, Australia

**Keywords:** brain tumor, deep learning, pattern detection, neuroscience, segmentation technique, convolution neural network, binary convolution neural network, magnetic resonance images

## Abstract

Neuroscience is a swiftly progressing discipline that aims to unravel the intricate workings of the human brain and mind. Brain tumors, ranging from non-cancerous to malignant forms, pose a significant diagnostic challenge due to the presence of more than 100 distinct types. Effective treatment hinges on the precise detection and segmentation of these tumors early. We introduce a cutting-edge deep-learning approach employing a binary convolutional neural network (BCNN) to address this. This method is employed to segment the 10 most prevalent brain tumor types and is a significant improvement over current models restricted to only segmenting four types. Our methodology begins with acquiring MRI images, followed by a detailed preprocessing stage where images undergo binary conversion using an adaptive thresholding method and morphological operations. This prepares the data for the next step, which is segmentation. The segmentation identifies the tumor type and classifies it according to its grade (Grade I to Grade IV) and differentiates it from healthy brain tissue. We also curated a unique dataset comprising 6,600 brain MRI images specifically for this study. The overall performance achieved by our proposed model is 99.36%. The effectiveness of our model is underscored by its remarkable performance metrics, achieving 99.40% accuracy, 99.32% precision, 99.45% recall, and a 99.28% F-Measure in segmentation tasks.

## 1 Introduction

Neuroscience is a rapidly evolving field dedicated to decoding the complex functions of the human brain and mind (Efford, 2000; Yamashita et al., 2018; Interpolation Methods, 2024).

Brain tumors represent a critical health challenge, potentially fatal at any stage of detection. A radiologist's knowledge and experience are vital when diagnosing brain tumors manually, yet it is not always accessible. Moreover, conventional diagnostic methods are time-intensive and susceptible to errors (Solanki et al., 2023). Brain cancer is a significant health concern, impacting individuals of any gender and at any life stage. A brain tumor consists of an aberrant growth of cells within the brain that proliferates differently and uncontrollably from normal brain tissues. To date, over 100 distinct types of brain tumors have been diagnosed. These tumors are broadly categorized into two main categories—primary and metastatic or secondary.

The tumor that develops inside or around the brain tissues is called a primary brain tumor. It can be benign (non-cancerous) or malignant (cancerous). Tumors that develop in other parts of the body and transfer or reach the brain are called secondary or metastatic brain tumors. Mostly, secondary brain tumors are considered malignant (cancerous). More than 84,000 persons in the United States were diagnosed with a primary brain tumor in 2021, according to the American Brain Tumor Association (ABTA). There are more than 100 subtypes of primary brain and central nervous system (CNS) cancers. Malignant tumors comprise about a third (29.7%) of all CNS malignancies. Currently, there are approximately 28,000 cases of pediatric brain tumors in the United States. Over 18,000 people lost their lives to primary malignant brain tumors in 2021. There is a significant difference in survival time after a diagnosis of a primary brain tumor based on factors such as age, region, race, tumor location, tumor type, and molecular markers (Oztek et al., 2023).

The World Health Organization (WHO) defines tumors in terms of grades—from Grade I to Grade I—based on their size and growth. Grade I and Grade II tumors are considered non-cancerous; they are slow-growing and curable. Grade III and Grade IV tumors are aggressive and grow very quickly. These types of tumors are considered malignant (cancerous) and categorized as metastatic or secondary tumors (Louis et al., 2021).

**Grade I**: Tumors in Grade I grow very slowly and do not spread aggressively. A patient with such a tumor can survive for a longer period; this tumor can be removed through surgery, and the patient can survive completely.

**Grade II**: Grade II tumors also grow slowly but can affect their neighboring tissues and progress to higher grades. After surgery, the tumor can develop again and affect the patient.

**Grade III**: Compared to Grade I and Grade II tumors, the rate of growth is faster in Grade III tumors, and it can affect the neighboring tissues quickly. Simple surgery is not very effective in removing this type of tumor; further post-surgery treatment is needed for survival.

**Grade IV**: These tumors are highly aggressive and spread to neighboring tissues. The blood vessels are the most important path for their growth. A patient diagnosed with such a tumor cannot survive for long.

Detecting brain tumors using deep learning, artificial intelligence, computer vision, and image processing has gained attention today. Automatic learning systems require different features to detect brain tumors, including shape, size, location, intensity, and growth. Researchers in the computer science field place a lot of importance on building robust and automated detection methods (Amin et al., 2021).

State-of-the-art research work has been done in the domain of brain tumor segmentation. Different methods have been proposed by researchers for the segmentation of brain tumors. One such method uses the convolutional neural network (CNN) to classify five types of tumors into three classes (Irmak, 2021). Another method based on the multiclass support vector machine (M-SVM) used meningioma, glioma, and pituitary brain tumors for segmentation. Yet another method was based on transfer learning for brain tumor segmentation (Maqsood et al., 2022). They used gliomas, meningioma, pituitary, and normal brain MRI images for brain tumor categorization. The model was trained on 75% of the images, and 25% were used for validation (Shoaib et al., 2022). However, gaps (Nida-Ur-Rehman et al., 2017; Irmak, 2021; Maqsood et al., 2022; Shoaib et al., 2022) still need to be filled while addressing brain tumor segmentation. Firstly, there are more than 100 types of brain tumors in the world that need to be segmented. Secondly, brain tumors are divided into four grades (Grade I to Grade IV) based on their size, growth, and aggressiveness. Each brain tumor must be accurately and timely classified into respective grades, which is very important. Thirdly, the image dataset used in brain tumor segmentation needs to be multivariant and multimodal to make the segmentation system more mature and accurate in brain tumor segmentation.

In this study, we propose a novel method that leverages deep learning using a binary convolutional neural network (BCNN) to classify the 10 most common types of brain tumors into their respective grades (Grade I to Grade IV); current models are limited to the detection of four brain tumor types. In our proposed model, image acquisition is followed by a comprehensive preprocessing phase, during which binary conversion using adaptive thresholding and morphological operations are executed. Secondly, segmentation is carried out to accurately classify tumor types into their respective grades. The model also accurately classifies healthy brain MRI images. Another contribution is the development of a dataset of 6,600 brain MRI images created for this research work; the dataset consists of different modalities, angles, and shapes for the entire brain model. The study is organized as follows: Section 2 includes a comprehensive discussion of brain tumor detection mechanisms. Our proposed model is explained in Section 3. Section 4 highlights the validation of the proposed technique using simulation experiments. Section 5 presents the conclusion and potential for future work.

## 2 Literature review

A detailed review of the latest and most relevant literature is presented in this section. An overview of the missing points in the literature is presented in a summary at the end of this section.

In Soomro et al. (2023), a detailed review of brain tumor segmentation from 1998 to 2020 is presented. It includes a complete overview of machine learning and image segmentation methods for brain tumor segmentation using MRI images. The state-of-the-art machine learning techniques and deep learning are reviewed, and at the end of their review, they establish that deep learning techniques perform better in brain tumor segmentation using MRI images.

Brain tumor segmentation and segmentation using MRI images detect many types of noise, including speckle noise, salt and pepper noise, and Gaussian noise, which may arise during the scanning process (Ramesh et al., 2021). Consequently, there may be lower accuracy rates in categorization. Therefore, the authors propose a novel noise-canceling algorithm—the iterative group median filter with modifications. Moreover, kernel principal component analysis based on maximum likelihood estimation is presented for feature extraction. The VGG16 architecture, which is based on deep learning, was used for the segmentation task. The suggested method has proven to perform better in both qualitative and quantitative experiment evaluations.

In Ahmed et al. (2016a,b), a total of 1,200 brain MRI scans of brain tissue damaged by tumors and 300 scans of normal brain tissue are included. The suggested approach is effective in detecting four different forms of brain tumors: CNS lymphoma, glioblastoma, meningioma, and metastases. This technique separates the tumor regions from healthy tissue using a 2D adaptive filter and Otsu segmentation. A combination of morphological operations and image fusion is used to highlight the tumor region so that it may be studied in detail.

In Nida-Ur-Rehman et al. (2017), brain tumors are broken down into four distinct categories. The dataset utilized in this study consists of two thousand magnetic resonance imaging (MRI) scans with a clinical and expert opinion from the FCPS neurosurgeon. Histogram differencing is utilized to segregate and detect tumor pixels from the rest of the brain tissues. The volume of data utilized in the categorization process might alter the final findings and cause them to differ between datasets.

In Le et al. (2021), the authors offer a deep learning methods-based strategy to detect and segment brain tumors. This investigation has two key phases. In the first phase, the network only pays attention to the area around the tumor to identify brain tumors using a contextual detection network. The #D atrous residual network is used in the second phase to segment tumors.

Comparative approaches of different segmentation techniques are used in Bahadure et al. (2018), and the best one is selected by comparing their segmentation score. Further, to improve the segmentation accuracy, the genetic algorithm is employed to automatically segment the tumor stage. The decision on the segmentation stage is supported by extracting relevant features and calculating the area. The experimental results of the proposed technique are evaluated and validated for performance and quality analysis on magnetic resonance brain images based on segmentation score, accuracy, sensitivity, specificity, and dice similarity index coefficient. The experimental results achieved 92.03% accuracy, 91.42% specificity, 92.36% sensitivity, and an average segmentation score between 0.82 and 0.93, demonstrating the effectiveness of the proposed technique for identifying normal and abnormal tissues from brain MRI images. The experimental results also obtained an average of 93.79% dice similarity index coefficient, which indicates better overlap between the automated extracted tumor regions and manually extracted tumor regions by radiologists.

In another study, researchers utilized a machine learning technique, specifically a Convolutional Neural Network (CNN) (Badža and Barjaktarović, 2020), for the segmentation of brain tumors. CNNs are well known performing high performance in image segmentation tasks. The authors introduced a novel CNN architecture tailored for segmenting three types of brain tumors. This new network is more straightforward compared to existing pre-trained models and tested using T1-weighted contrast-enhanced MRI scans. The network's performance is assessed through four different methods: two variations of 10-fold cross-validation and two distinct databases. Its ability to generalize is evaluated using subject-wise cross-validation, and improvements are measured with an augmented image database. The highest accuracy, 96.56%, is achieved with record-wise cross-validation on the augmented dataset. With its robust generalization and swift execution, this CNN architecture shows promise as a decision-support tool for radiologists in medical diagnostics.

In Garg and Garg (2021), a method is evaluated using a dataset of 2556 images, split 85:15 for training and testing, achieving an accuracy of 97.305%. This method involves brain tumor segmentation using a hybrid ensemble approach that combines K-Nearest Neighbors (KNN), Random Forest (RF), and Decision Tree (DT) based on the Majority Voting method, namely KNNRF-DT. The aim is to calculate the tumor region's area and classify tumors as benign or malignant. Otsu's Threshold method is used for segmentation, while feature extraction is performed using Stationary Wavelet Transform (SWT), Principal Component Analysis (PCA), and Gray Level Co-occurrence Matrix (GLCM), providing thirteen features for segmentation. The hybrid ensemble classifier (KNN-RFDT) based on Majority Voting aims to enhance the performance of traditional classifiers without resorting to deep learning techniques.

In Phan and ThanhHieu (2024), a combination of three different existing algorithms is proposed for segmenting brain tumors. The algorithms used are the PGDBCWMF algorithm for noise removal in the preprocessing, the SIFT (scale-invariant characteristic remodel) approach for feature extraction, and the HV region algorithm for segmenting brain tumors. A brain and pancreatic tumor dataset is used for the segmentation of tumors.

In Akter et al. (2024), a deep convolution neural network is proposed for the classification and a U-NET-based segmentation model for the segmentation of four different categories of MRI images. The four different categories consist of glioma brain tumor, pituitary brain tumor, meningioma brain tumor, and images with no tumor. Six different datasets were used to train the segmentation model and to test the classification model. The overall accuracy achieved by their proposed model is 98.7% based on the merged dataset, 98.8% accuracy in the segmentation section, and 97.7% classification accuracy with individual datasets.

The literature review reveals numerous missing aspects and requires attention to create a more robust and accurate segmentation module. Firstly, very few brain tumor types have been considered for segmentation. Secondly, tumor types must still be classified into grades from Grade I to Grade IV. Finally, the dataset of images needs to be multivariate and multimodal and offer diverse features so that the segmentation module classifies every image easily and accurately.

## 3 Materials and methods

### 3.1 Dataset of brain MRI images

The dataset of brain MRI images used in this study is collected from Nida-Ur-Rehman et al. (2017) and Radiopaedia's (2023). The dataset contains brain MRI images of 10 tumor types and healthy brain MRI images (Table 1). The collected dataset of images was checked and verified by doctors from the medical field for its authenticity. The dataset included multimodal and multivariant brain MRI images to cover all the angles, shapes, and positions of the brain for the classification of tumors. Table 1 presents the number of MRI images used for each type of tumor and healthy brain MRI images.

**Table 1:** Dataset of brain MRI images of each tumor type and healthy brain MRI images.

**S. No**	**Tumor types**	**Number of MRI images**
1	CNS Lymphoma	800
2	Glioblastoma	600
3	Meningioma	600
4	Metastases	400
5	Astrocytoma	600
6	Cystic Pituitary Adenoma and Meningioma	500
7	Ependymomas	600
8	CNS Embryonal Tumor NOS	500
9	Oligodendrogliomas	500
10	Hemangioblastomas	500
11	Healthy brain MRI images	1,000
Total		6,600

**CNS Lymphoma**: Primary central nervous system Lymphoma is a type of brain tumor that can be primary and secondary; in this type of tumor, cells emerge in the lymphoma and/or the spinal cord region (RMH Neuropathology, 2013).

**Glioblastoma**: This type of tumor is considered dangerous because it grows fast and spreads quickly inside the brain. Initially, glioblastoma attacks adjacent brain tissues (Gaillard, 2018).

**Meningioma**: This brain tumor starts inside the brain tissues called meninges that protect the brain and spinal cord. Most meningiomas are not dangerous but can reach up to Grade III tumor levels (Di Muzio, 2023).

**Metastases**: These types of tumors spread from other parts of the body, such as the lungs, breasts, and kidneys, to the rest of the body. Once they spread to the brain, they can create one or more tumors inside the brain (Brusic, 2021).

**Astrocytoma**: These tumors can be cancerous or non-cancerous. Some grow very slowly, while others can be aggressive. They appear first in cells called astrocytes (Gaillard, 2021).

**Cystic Pituitary Adenoma and Meningioma**: They are generally slow-growing types and fall in the benign category of brain tumors, which are mostly considered Grade 1 or Grade II tumors. Most patients with this type of tumor are diagnosed after several years before observing any signs. It develops from pituitary tissues and grows inside the pituitary gland of the brain (Gaillard, 2016).

**Ependymomas**: This type of brain tumor develops inside the brain or spinal cord area. It can reach Grade 3 from Grade 1. It initially begins in ependymal cells that help to maintain and improve brain streams (Schubert, 2011).

**CNS Embryonal Tumor NOS**: It is the most common type of brain tumor found in children <3 years of age. By nature, this type of tumor is malignant, and it exists in the area of the cerebellum as a solid mass (Jones, 2021).

**Oligodendrogliomas**: They emerge around the brain and cortex, the brighter white portion of the brain. They are most commonly considered the middle-aged adult's tumor (Gaillard, 2010).

**Hemangioblastomas**: They are benign brain tumors that mostly rise around the brain, spinal cord, and behind the eye tissues (retina). They normally occur in young and middle-aged people. (Balachandran, 2010).

**Healthy brain MRI images**: An MRI image without any diagnosis of a brain tumor. These brain MRI images are considered to have no tumor signs present. The shape, nature, and performance of the brain cells are normal in the healthy brain MRI images. There is no sign of abnormality, nor are any abnormal tissues found inside the healthy brain MRI images.

**Figure 1:**
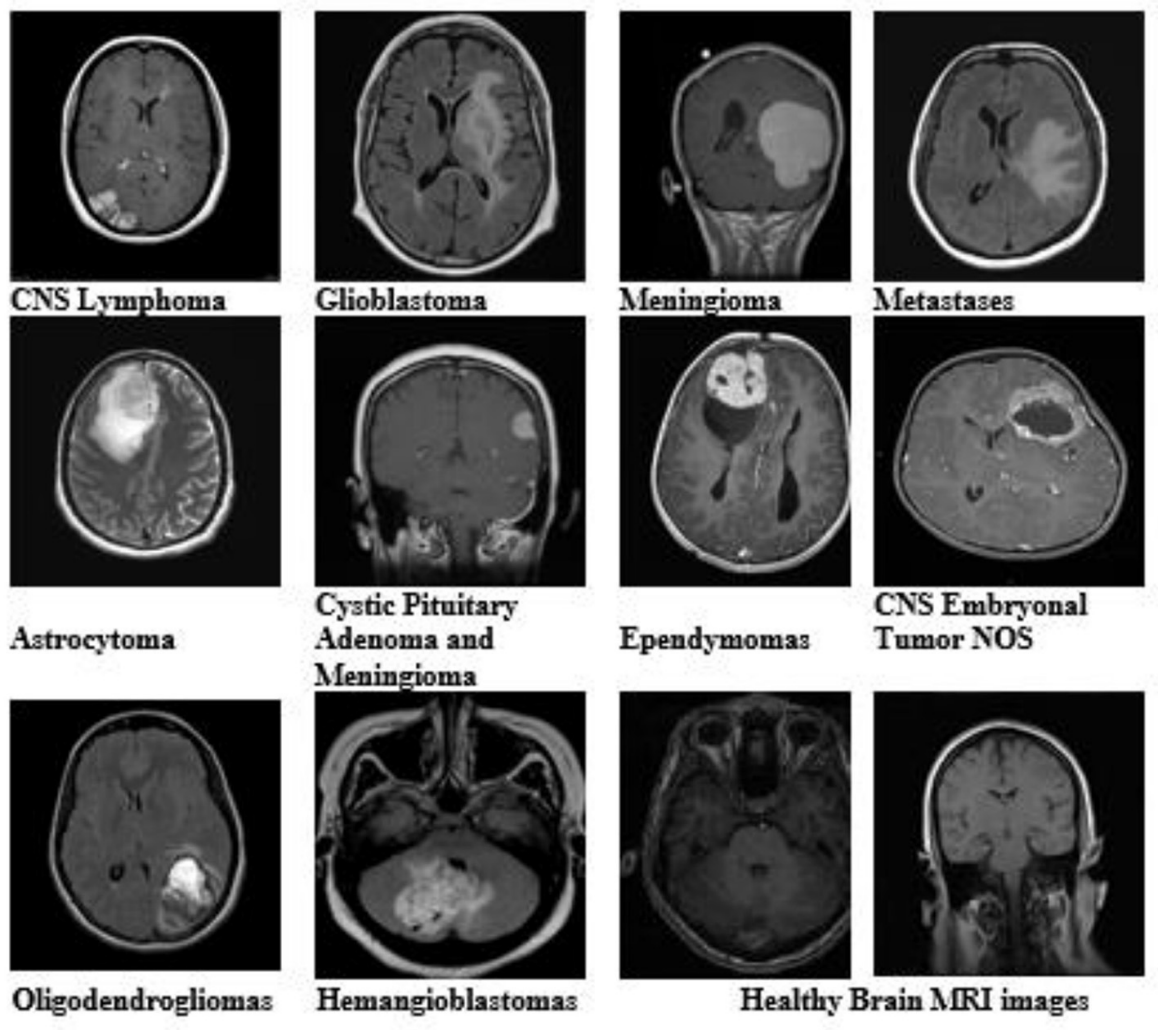
Brain MRI images of all tumor types and healthy brain MRI images from the dataset with different variants and modalities.

Figure 1 shows a sample of images from the brain MRI dataset, including healthy brain MRI images.

### 3.2 Research methodology

Brain tumors exhibit distinct variations in size, intensity, and contrast compared to normal tissue in MRI scans, which are critical for tumor classification. Leveraging deep learning, which performs complex computations on vast datasets through artificial neural networks, mimics human cognitive processes for automated learning.

In this study, we propose a binary convolutional neural network (BCNN) tailored for the segmentation of various brain tumor types in MRI imagery. BCNNs, characterized by multiple processing layers, excel in extracting features from extensive data, making them ideal for detailed feature analysis within large datasets.

Our methodology unfolds in two stages. In the first stage, image acquisition and preprocessing are conducted, where images are resized and their intensity or contrast levels adjusted. Subsequent steps involve converting these preprocessed images into binary format using adaptive thresholding, followed by morphological operations to delineate and classify the tumor regions by grade.

**Figure 2:**
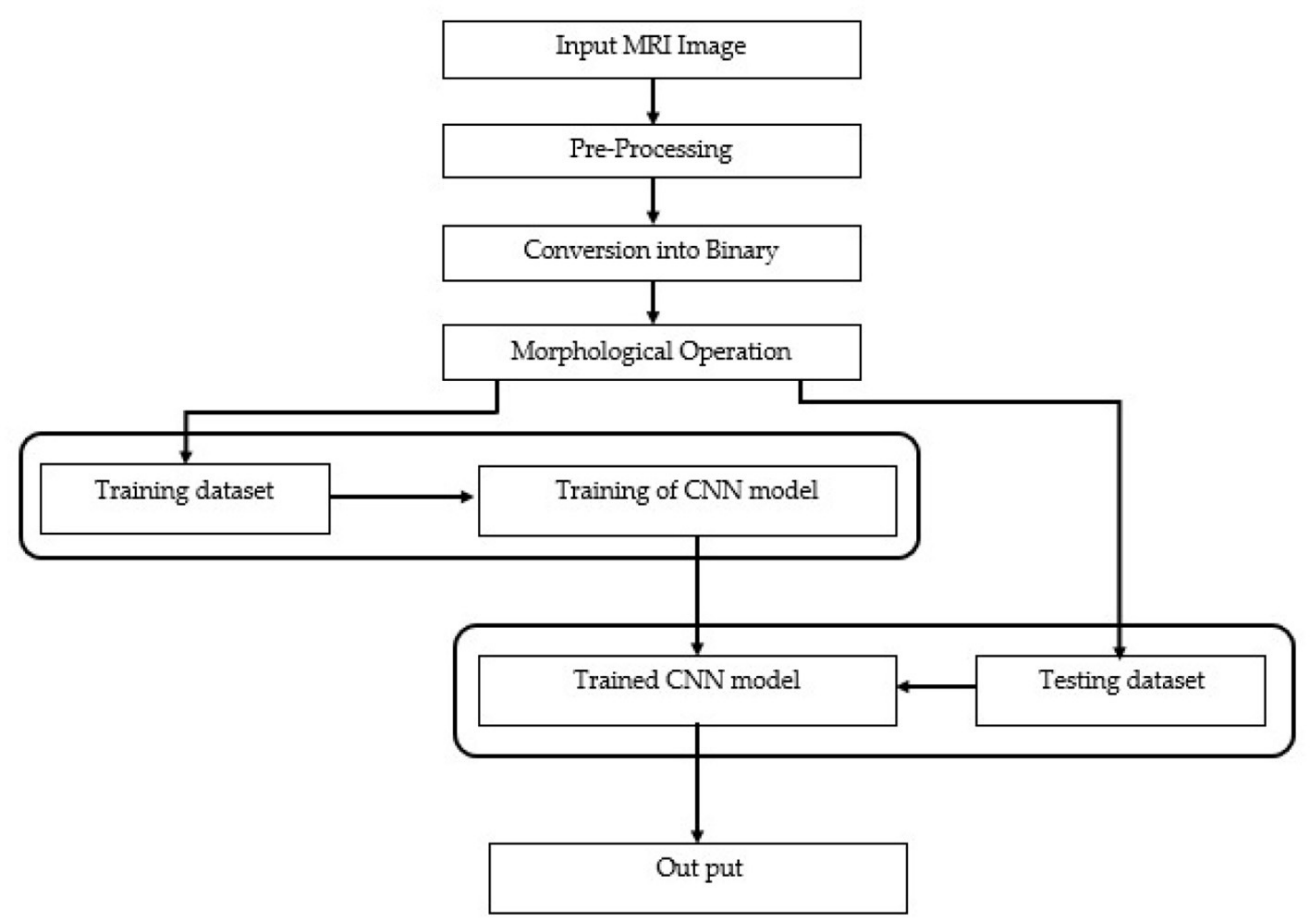
A step-by-step flow diagram of the proposed research methodology.

In the second stage, the preprocessed images, now segmented, undergo further classification into specific tumor grades, from Grade I to Grade IV, utilizing the size characteristics of the tumor regions. The data generated through preprocessing serves as the foundation for training and testing our BCNN model. Approximately 80% to 90% of our labeled dataset, encompassing both tumor-afflicted and healthy brain MRI scans, is allocated for model training. The remaining 10% to 20% of segmented but unlabeled data is reserved for model testing, assessing the BCNN's efficacy in classifying the data into correct tumor grades or identifying healthy tissue. The data is labeled initially with the support of experts from the medical field, especially a senior FCPS neurosurgeon. The labeling processing with the help of medical experts gives us a clear view and understanding of tumor size, growth, grades, and different modalities and variations of brain tumor and MRI images. Ultimately, the output will distinguish between accurate (true segmentation) and inaccurate (false segmentation) classifications, with the model striving to precisely categorize each MRI scan into the appropriate tumor grade or as a healthy brain image. The comprehensive flow of these phases is depicted in Figure 2, illustrating each step from initial preprocessing to final classification in our proposed research methodology. Using this methodology, we accurately segment brain tumors from healthy brain MRI images and also classify the ten most common types of tumor into their respective grades (Grade 1 to Grade IV).

#### 3.2.1 Preprocessing

The MRI images are captured under different light conditions, and their sizes differ. Therefore, during preprocessing, we convert the images into equal sizes, normalize the lighting effect, or set the contrast of the image. This will help us convert an image into a perfect condition to get more accurate results during segmentation. We convert each image to an equal size of 600^*^600 pixels dimensions. The resizing of the image uses the nearest-neighbor interpolation. Once we give an image to the algorithm, Matlab converts the image automatically into the defined dimensions of 600^*^600.

##### 3.2.1.1 Image resizing—Nearest-neighbor interpolation

Nearest-neighbor interpolation is a simple yet effective method for quickly converting grayscale images into different sizes as required. In this method, the known value of the nearest pixel is taken without paying attention to other pixels.

**Figure 3:**
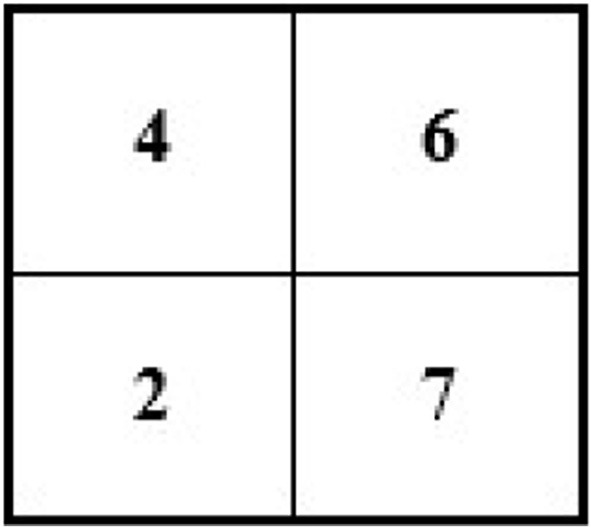
Showing 2 × 2 size original image with the pixel value.

For instance, an image of 2 × 2 pixels (see Figure 3) can be converted and its size maximized to 4 × 4 pixels (the size of the image grid becomes 4 × 4 pixels).

**Figure 4:**
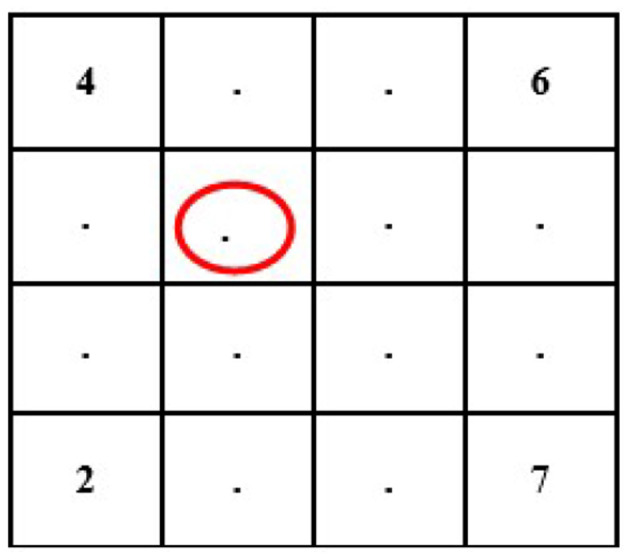
Showing 4 × 4 size images with unknown pixel values.

For example, we have an image of the following size and pixels:

In this case, we know the value of a few pixels in 2 × 2, which is then converted into 4 × 4 to interpolate other unknown pixels (red circle) (see Figure 4).

**Figure 5:**
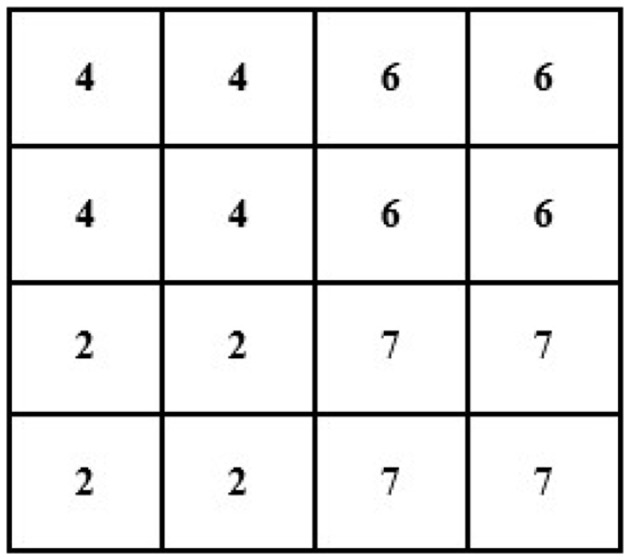
Showing 4 **×** 4 size images after interpolation of known pixel values.

Now, we will need to find the value of an unknown pixel in the red circle. The nearest neighbor value of the unknown pixel value in the red circle in Figure 4 is 4, which is known, so the value of the unknown pixel in the red circle will become 4. Similarly, the remaining unknown pixel values will be filled out in the same fashion (see Figure 5).

**Figure 6:**
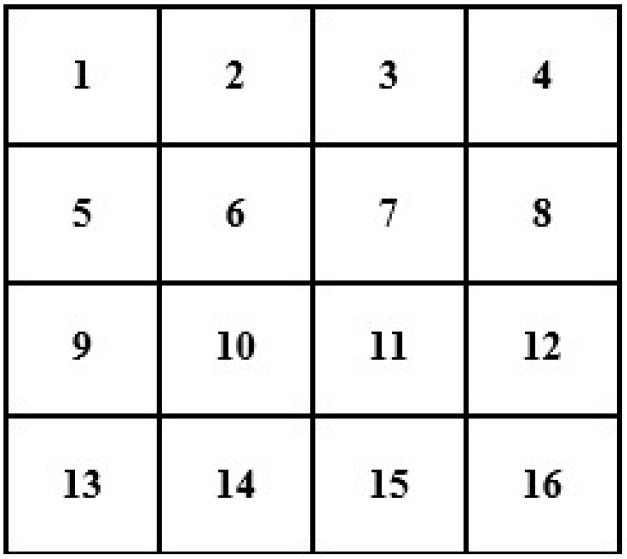
Showing original 4 **×** 4 size image with known pixel values.

If we have an image of 4 × 4 and want to minimize its size to 2 × 2, then the new size of the image will become 2 × 2 (see Figure 6).

**Figure 7:**
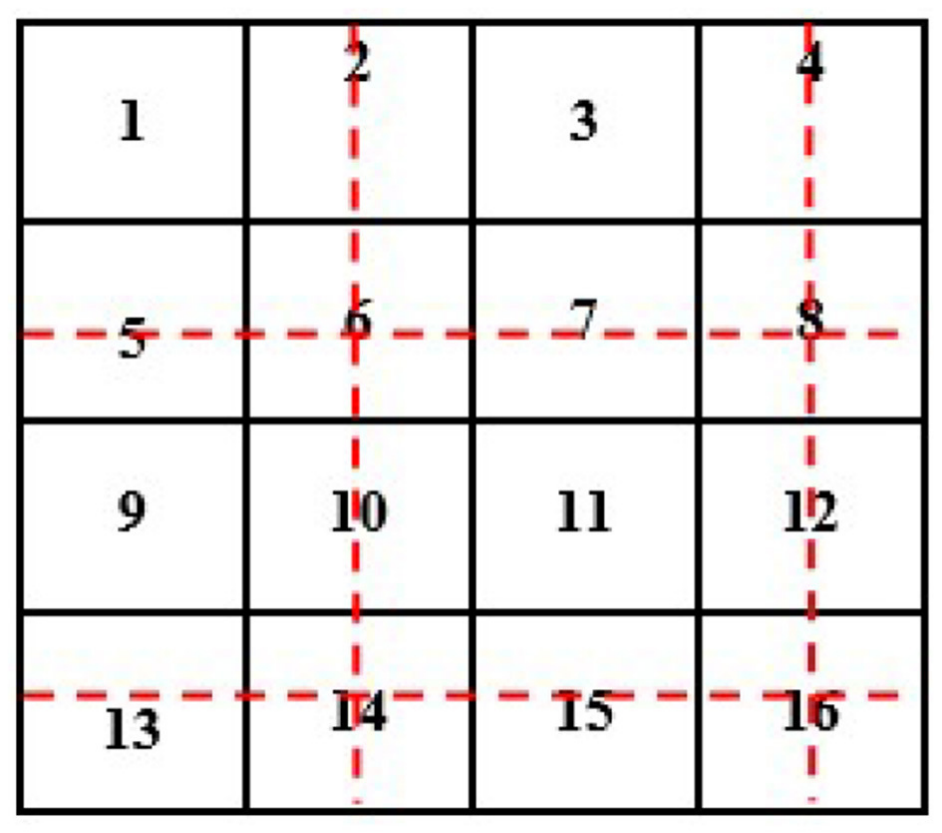
Showing 4 **×** 4 original size image conversion into 2 **×** 2.

To reduce the size of the above image from 4 × 4 to 2 × 2, we remove every second row and second column (see Figure 7).

**Figure 8:**
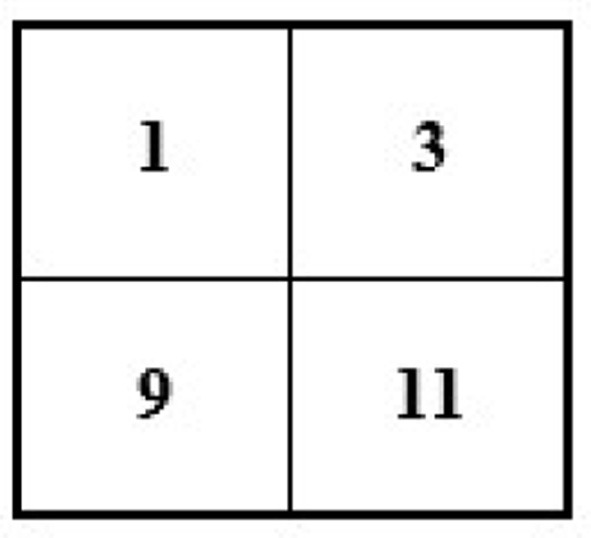
Showing 2 **×** 2 size image after interpolation/conversion from 4 **×** 4.

After removing the second row and second column, we will have the following image with a 2 × 2 size (see Figure 8).

##### 3.2.1.2 Image filtering using two-dimensional adaptive filter

The two-dimensional adaptive filter estimates the local mean and variance around each pixel. It reduces the mean square error as much as possible [see Equation (1) and Equation (2)].


(1) $$\begin{array}{l} \mu =\;\frac{1}{NM}\underset{n_{1}{,n}_{2}\;\in \;\eta }{\sum }a\left(n_{1},n_{2}\right) \end{array}$$

(2) $$\begin{array}{l} \sigma^{2}=\frac{1}{NM}\underset{n_{1}{,\;n}_{2}\;\epsilon \;\eta }{\sum }a^{2}\left(n_{1}{,n}_{2}\right)-\mu^{2} \end{array}$$

Where the N-by-M local neighborhood of each pixel is in image *A*, the two-dimensional adaptive filter creates a pixel-wise Wiener filter using these estimates [see Equation (3)].


(3) $$\begin{array}{l} b\left(n_{1},n_{2}\right)=\;\mu +\;\frac{\sigma^{2}-\nu^{2}}{\sigma^{2}}\left(a\left(n_{1},n_{2}\right)-\mu \right) \end{array}$$

Where **ν**^**2**^ is the noise variance. If the noise variance is not given, the two-dimensional adaptive filter uses the average of all the local estimated variances.

##### 3.2.1.3 Conversion into binary and morphological operation

Once the preprocessing step is completed, the image is converted into binary, and the morphological operations are performed. The grayscale image is converted into binary using the adaptive thresholding method. A threshold value is calculated locally using the mean of the neighborhood pixels using a filter; if the pixel value is above the threshold, it will be considered a foreground value or one; otherwise, it will be considered a background value or zero. In this method, a mean filter around the neighborhood is subtracted from a constant value of the pixels to find the foreground pixels.

For example:

*T* is our threshold value for the output image, *M* is set to be our threshold value for the mean filter of the neighborhood, and *C* is our constant value to be subtracted from *T*. Ultimately, it will give us a new binary image with foreground values as our resultant image (Equation 4).


(4) $$\begin{array}{r} T\;threshold=M\;mean\;of\;neighboorhood\;pixels-\\ C\;constant \end{array}$$

Morphological operations remove any noise or unwanted pixels that may cause errors or produce false segmentation results. The binary-converted image shows two types of pixels—black and white. The white pixels show the foreground pixels or the targeted pixels, which include the tumor area and some other extra and unwanted noisy pixels, such as the boundary of images. We need to remove the extra and unwanted noisy pixels to focus attention on the tumor pixels. This will help us keep the tumor pixels as foreground pixels and use them further during segmentation.

**Figure 9:**
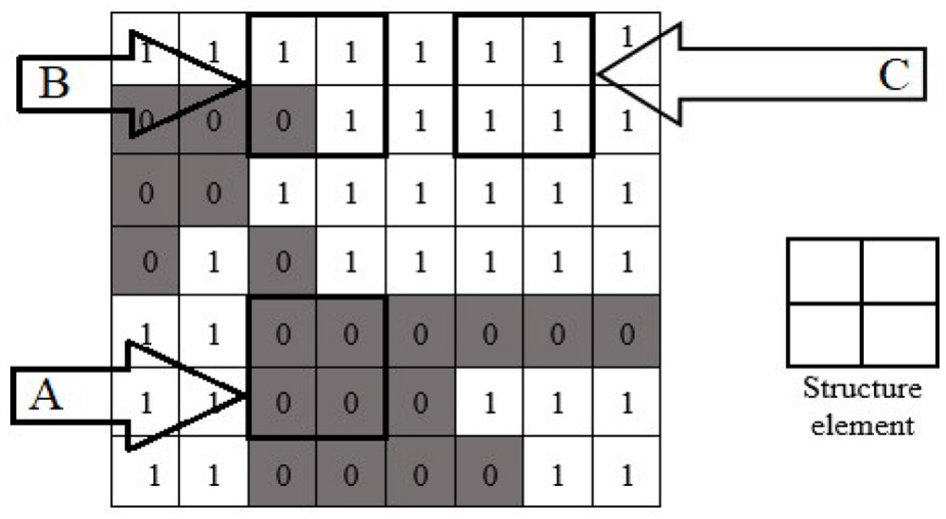
Morphological operation's structure element with fit **(A)**, hit **(B)**, and neither fit nor hit **(C)** the image.

Morphological image processing is a collection of non-linear operations related to the shape or morphology of features in an image. Morphological techniques probe an image with a small shape or template called a structuring element. The structure element is a pre-defined matrix or binary image with values 0 and 1, which is used to probe the image. The structuring element is positioned at all possible locations in the image and compared with the corresponding neighborhood of pixels. Some operations test whether the element “fits” within the neighborhood, while others test whether it “hits” or intersects the neighborhood. The actual structure element that we used for erosion and dilation was 22^*^22. An example of using a structure element can be seen in Figure 9.

When a structuring element is placed in a binary image, each of its pixels is associated with the corresponding neighborhood pixel under the structuring element. The structuring element is said to fit the image if, for each pixel set to 1, the corresponding image pixel is also 1. Similarly, a structuring element is said to hit or intersect an image if, at least for one of its pixels set to 1, the corresponding image pixel is also 1. In Figure 9, the structure element is the design of 2 × 2. The structure element fits at location “A” because the structure elements of 2 × 2 all fit the position “A” of the image. The structure element hit the image at position “B.” The structure element neither hit nor fit at position “C.”

The erosion of a binary image *f* by a structuring element *s* (denoted *f* ⊖ *s*) produces a new binary image *g* = *f* ⊖ *s* with ones in all locations *(x, y)* of a structuring element's origin at which that structuring element *s* fits the input image *f*, i.e., *g(x, y)* = *1* is *s* fits *f* and *0* otherwise, repeating for all pixel coordinates *(x, y)*. The erosion shrinks an image by removing a layer of pixels from the inner and outer boundaries of image regions. The holes and gaps between different regions of the image become larger, and small details or noise get eliminated. Erosion removes small-scale details from the binary image but simultaneously reduces the size of regions of interest. We perform a dilation operation in the morphology to maintain the foreground pixels or the tumor pixels. It adds the region of tumor pixels removed in the erosion stage. The dilation of an image *f* by a structuring element *s* (denoted *f* ⊕ *s*) produces a new binary image *g* = *f* ⊕ *s* with ones in all locations *(x, y)* of a structuring element's origin at which that structuring element *s* hits the input image *f*, i.e., *g(x, y)* = *1* if *s* hits *f* and *0* otherwise, repeating for all pixel coordinates *(x, y)*. Dilation has the opposite effect of erosion—it adds a layer of pixels to both the inner and outer boundaries of the regions.

##### 3.2.1.4 Binary convolution neural network—BCNN

After the binarization and morphological operations, we used BCNN as the main part of our methodology. The BCNN is used to classify the tumor of every tumor type used in this study into grades. Because BCNN works on the binary images generated from the morphological steps, all the binary images generated from the morphological steps are saved in different folders labeled with tumor grades (from Grade I to Grade IV) and the healthy brain MRI images folder. Labeling all the folders of binary images from Grade I to Grade IV tumors and healthy brain MRI images is for the training of the BCNN.

The BCNN stores values in binary formats 1 and 0. This process, known as 1-bit quantization, saves memory, increases the processing speed of the network, and reduces memory access time. Overall, it is fast in computation and uses less memory. The binary images or data used to train our neural network are most suitable for embedded and microcontroller devices.

The general weights of CNN depend on grayscale and color images, which have three values. In our BCNN, a binarization function is used to binarize those values. The two functions used are sign and stochastic [see equations (5–7)].


(5) $$x^{b}=Sign\left(x\right)=\{\begin{array}{c} +1\;if\;x\;\geq 0,\\ -1\;otherwise, \end{array}$$

(6) $$\begin{array}{l} x^{b}=\begin{array}{c} +1\;with\;probability\;p=\sigma \left(x\right),\\ -1\;with\;probability\;1-p,\; \end{array} \end{array}$$

(7) $$\begin{array}{l} \sigma \left(x\right)clip\left(\frac{x+1}{2},0,1\right)\max\left(0,\min\left(1,\;\frac{x+1}{2}\right)\right) \end{array}$$

**Figure 10:**
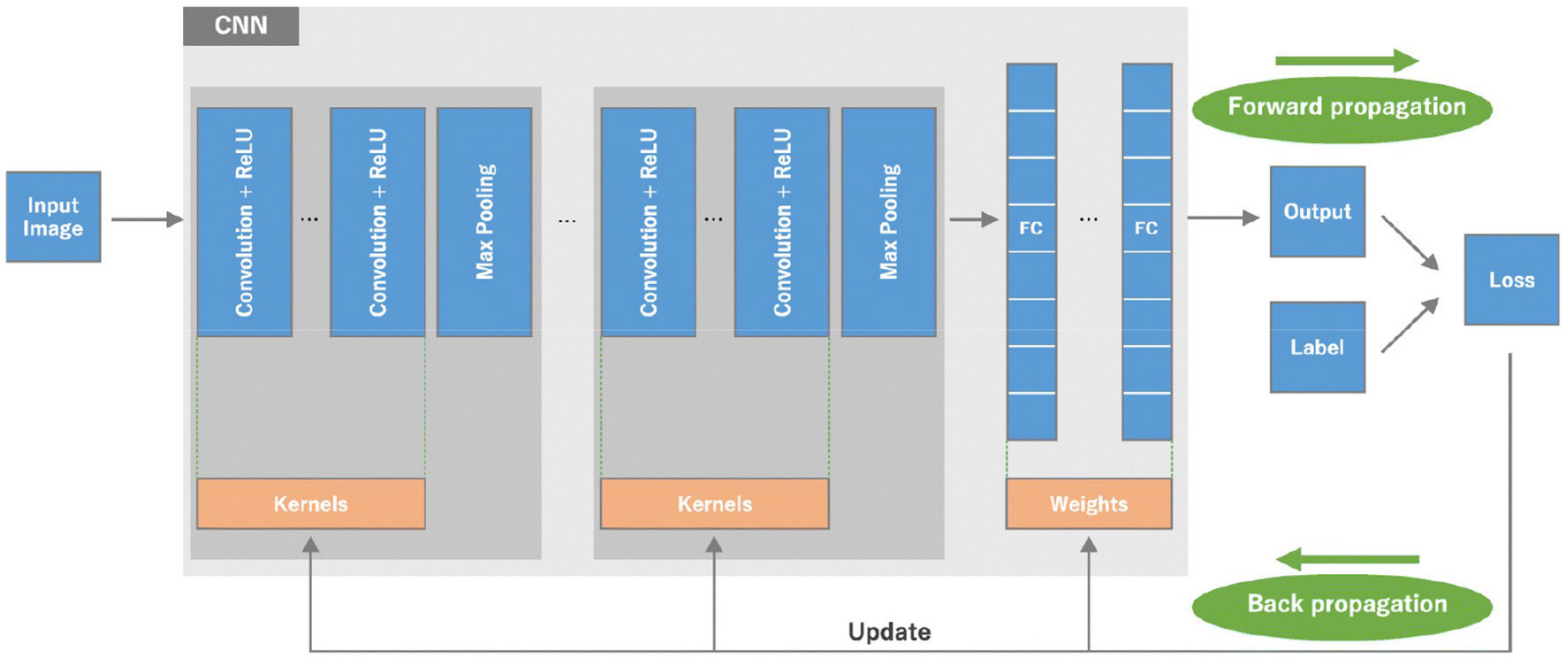
The basic structure of the neural network.

Our BCNN has three main convolution layers and one fully connected layer. The three convolution layers include the input convolution layer, pooling layer, and batch normalization layer. The basic structure of the neural network can be seen in Figure 10.

The three main convolution layers and one fully connected layer that are used to build our BCNN are discussed in detail below.

A. **Convolution layer**

**Figure 11:**
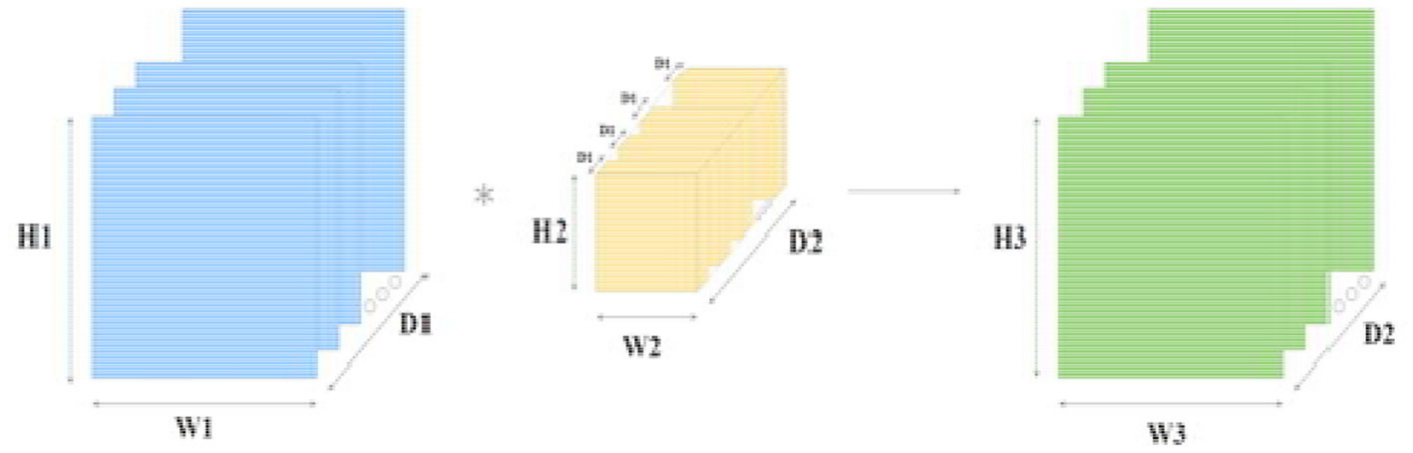
Convolution layer with input, kernel, and output layer; blue is the input to the convolution layer, yellow is the kernel (filter), and green is the output of the convolution layer.

The convolution layer is the first layer used in a BCNN. It gets the input matrix of dimensions, which includes the elements H1 x W1 x D1; H1 is the height of the matrix, W1 is the width of the matrix, and D1 is the dimension of the matrix. Next, we have kernels (structure elements or filters) in the convolution layer. A kernel is a matrix with dimensions H2 x W2 x D2. A convolution layer has multiple kernels placed on top of each other in a sequence. These multiple kernels above each other create a 3-dimensional matrix; D2 is the number of dimensions. At the end of the convolution layer is the output layer with the dimensions H3 x W3 x D2. A detailed representation of the three sections of the convolution layer is presented in Figure 11.

B. **Pooling layer**

The pooling layer minimizes features of the in-plane dimensionality to make a new invariance to small changes and misrepresentations and minimize the upcoming parameters. The advantage of using a max pooling layer is that it minimizes the number of parameters of the input plot and minimizes overfitting, extracts important features from the input plot, minimizes computation, and, therefore, introduces maximum efficiency. In the pooling layer, there are no such learnable parameters. Filters, padding, and strides are used as hyperparameters in the pooling layer, similar to convolution layers.

**Figure 12:**
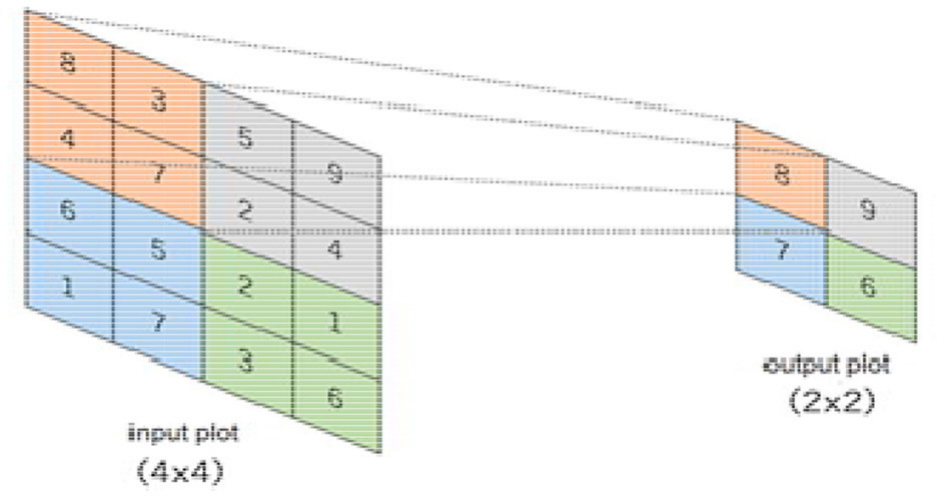
Showing pooling method functionality.

The max pooling method is used in our pooling layer in BCNN. The max pooling operation is used to extract patches from the input features plot, and an output produces a new plot extracting the maximum number in each plot and discards the rest of the values. Other than height and width, this will not change the depth dimension of the feature map. Figure 12 presents a 4 × 4 input plot while extracting a new 2 × 2 plot, extracting the maximum value in each plot by using a 2 × 2 filter on the input plot.

C. **Batch normalization layer**

Before training, batch normalization is used as a preprocessing step in the neural network. It improves the learning capability of the network and also avoids overfitting. It converts the data into a standard format before the training phase of the network to make the training phase easy. Since we have different types of MRI images in terms of size, shape, and intensity, it would be challenging for the network to train on such diverse data. Moreover, this would make the network more complicated and less efficient, and we may not be able to learn 100% as per our target. Consequently, the overall capability of the network would be decreased.

The approach we use inside the batch normalization layer is to scale it to a selection from 0 to 1. In Equation 7, *x* is the facts factor to normalize, *m* is the mean of the data set, *x*_*max*_ is the maximum value, and *x*_*min*_ is the minimum fee. This technique is normally used in data input. The non-normalized data inputs with huge variations can produce instability in neural networks. The tremendously big inputs can cascade down to the layers, inflicting problems that include exploding gradients [see Equation (8)].


(8) $$\begin{array}{l} x_{normilization}=\frac{x-m}{x_{\max}-x_{\min}} \end{array}$$

D. **One fully connected layer**

It works as a feed-forward layer in the neural network. It receives input from the previous pooling and batch normalization and is forwarded for further processing. It is a unidirectional layer that receives input from one direction and forwards in the same direction without using any repetition or loop. The input received by the fully connected layer from the previous layers is in a vector format. The fully connected layer has hidden layers, which are a combination of affine and non-linear functions. The one affine and one non-linear layer is called one fully connected layer or one hidden layer. We can add additional fully connected layers or hidden layers as per the requirement of our segmentation model.

The calculation in Equation (9) is used for every fully connected layer of the neural network. In Equation (9), *x* represents the input from the previous layer as the input vector, *w* is the weight matrix with dimensions, *b* is the bias vector, and *g* is the activation function, usually ReLU [see Equation (9)].


(9) $$\begin{array}{l} g\left(wx+b\right) \end{array}$$

**Figure 13:**
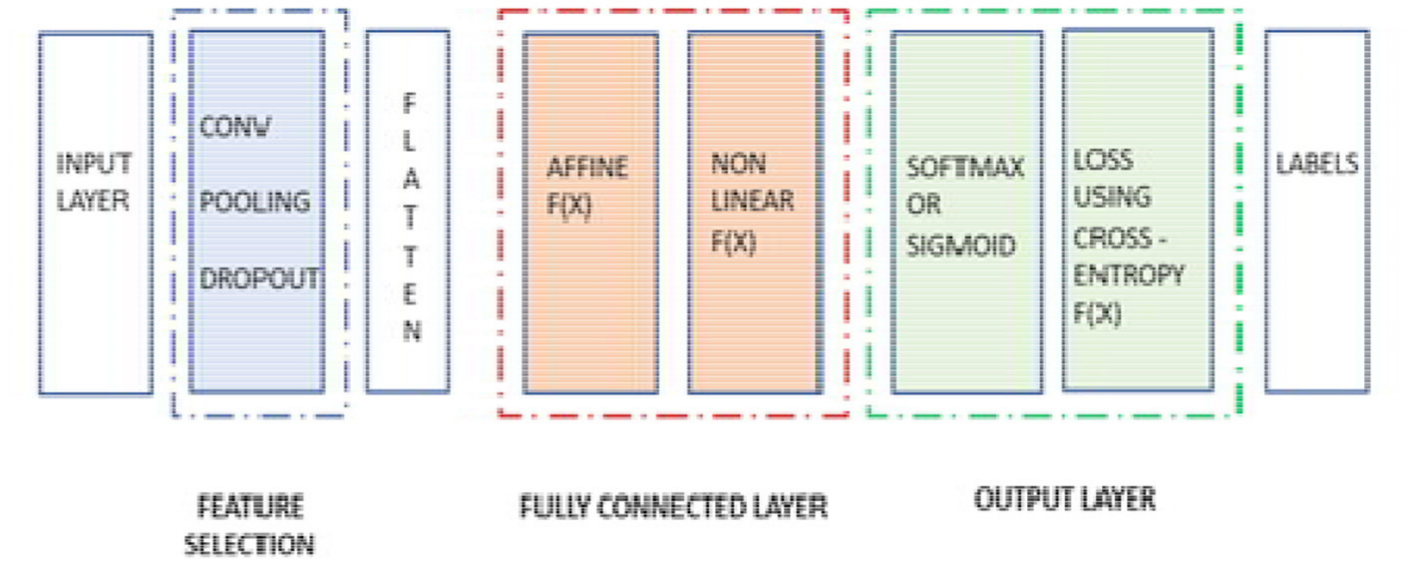
Different sections of the neural network.

After the process completion of the fully connected layer, and once it passes from the last layer, it is used to calculate the probability and classify the values into their respective class. Finally, we get the probability of the object or input data in the class to which it belongs. That is how the overall neural network works. The mechanism of the neural network is displayed in Figure 13, where the pooling and batch normalization work as feature selection layers, and the rest of the section of the neural network is used as a part of the segmentation layer, which includes a flattening layer, a fully connected layer, and the final output layer.

## 4 Results and discussion

This section discusses the complete step-by-step results of our proposed research work. The software used for the implementation and analysis of our proposed research methodology is also explained in this section.

We used Matlab version 2021 software in our research to implement our proposed methodology and analysis. Matlab provides state-of-the-art functionality and facilitation to easily implement and analyze complex and tricky methodologies. We used an HP Core-i5 7th generation computer/laptop to run the software and generate the overall results. The detailed implementation and the results of our proposed research work are discussed below.

The proposed methodology is implemented in two phases. In phase one, preprocessing, conversion into binary, and morphological operations are applied to the input images. In the second phase, the tumor types are segmented into grades.

### 4.1 Implementation and results of phase one

#### 4.1.1 Step one

**Figure 14:**
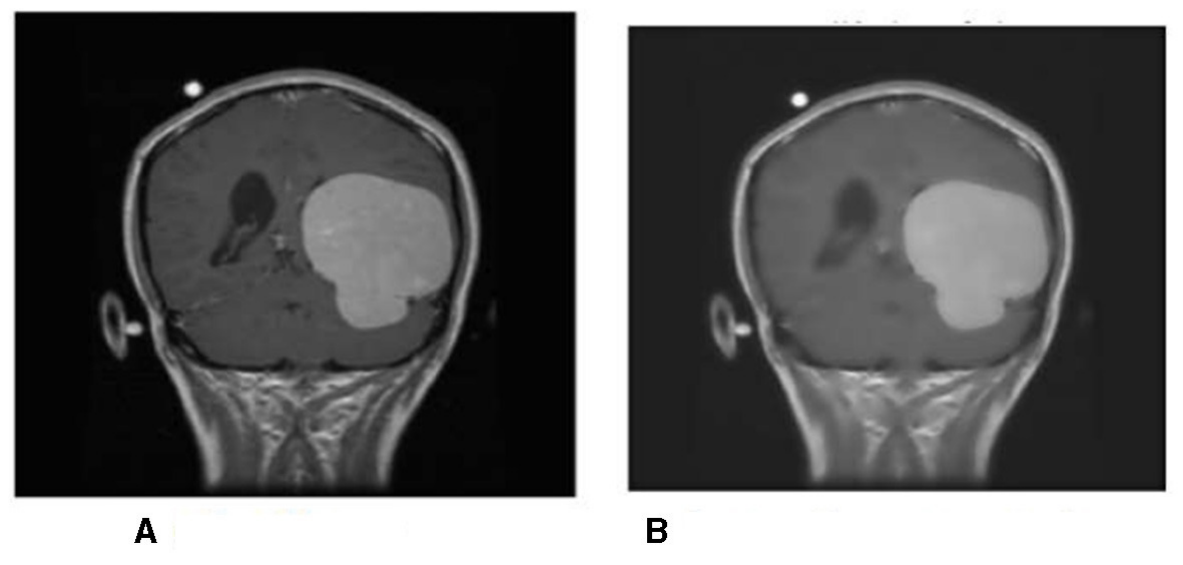
**(A)** The original image from the data set; **(B)** The resultant image after preprocessing.

First, the brain tumor MRI image from the dataset is passed to the first step of our methodology, which is preprocessing. The preprocessing step is performed to remove any unwanted impurities, enhance the quality of the image, and convert all the images to a standard and equal size. Performing the preprocessing step provides better results at the end of the segmentation. The images we received from the two sources differed in size, intensity, and lighting. The preprocessing step is performed to make the images more useful. The difference in the original image (a) and the resultant image (b) after preprocessing is shown in Figure 14.

The intensity and size of both the images are different. The original image size is 812^*^812 pixels, and the resultant image size is 600^*^600 pixels. Preprocessing is performed to decrease or increase the size of the image to a standard size of 600^*^600 pixels.

#### 4.1.2 Step two

In step two, the resultant image is further processed and converted to binary format, and morphological operations are performed; the conversion into binary format is undertaken to make the segmentation phase more efficient and faster. This step is performed to identify the tumor region and remove the boundaries or skull region to easily classify the tumor region. Once the image is converted to binary, morphological operations such as erosion and dilation are performed. Once the preprocessing resultant image is converted into binary, then there are only two pixel values, 0 and 1, left to deal with; 0 represents the background pixels or of the image which are not our targeted pixels, and 1 represents foreground pixels which are our target pixels. These pixels represent the tumor pixels and the boundaries and extra foreground pixels of the image that need to be removed to retain only the tumor foreground pixels.

**Figure 15:**
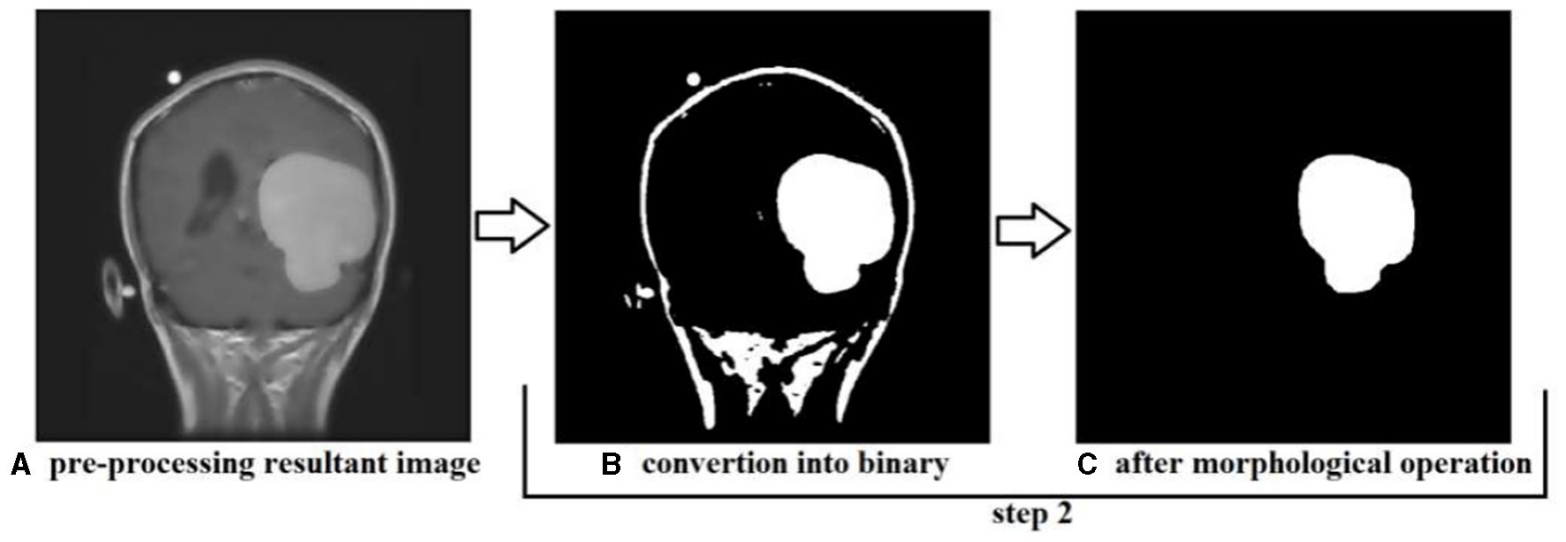
Showing the results produced after performing step 2, **(A)** is the resultant image of step 1, **(B)** is the resultant image converted into binary, and **(C)** is the image after performing morphological operation erosion and dilation.

Applying the erosion removes the extra or unwanted foreground pixels. This process also removes the boundaries of the skull in the image and some other parts. The erosion also affects the tumor foreground pixels, which affects the exact size of the tumor. To regain the actual size of the tumor size after erosion, we perform dilation; with the help of dilation, the tumor area that is removed or affected during erosion gets added to the tumor region again. This helps us to maintain the tumor region's original size and get accurate results in the segmentation. The results produced during step two of conversion into binary and morphological operations are shown in Figure 15. We can see that the tumor region in Figure 15A, which is the resultant image of step 1, can also be seen in Figure 15C after performing morphological operations.

### 4.2 Implementation and results of phase two

In step two, the actual segmentation of the tumor types into grades is performed. To start the segmentation, we first labeled all the resultant images produced after the morphological operation of step two. All the images were stored in different folders according to the grades of the tumor; the folders were labeled Grade I Tumor, Grade II Tumor, Grade III Tumor, Grade IV Tumor, and Healthy Brain MRI image.

Labeling is performed to train our BCNN segmentation model. The model will train on the images stored in binary format in all the labeled folders. All the images stored in the labeled folders are in binary format. Approximately 90% of the resultant images processed from our actual brain MRI images are used to train our segmentation model. The segmentation model is trained to classify tumor types into their respective grades.

The actual performance of our proposed segmentation model includes the following steps:

#### 4.2.1 Preparation of labeled data

**Figure 16:**
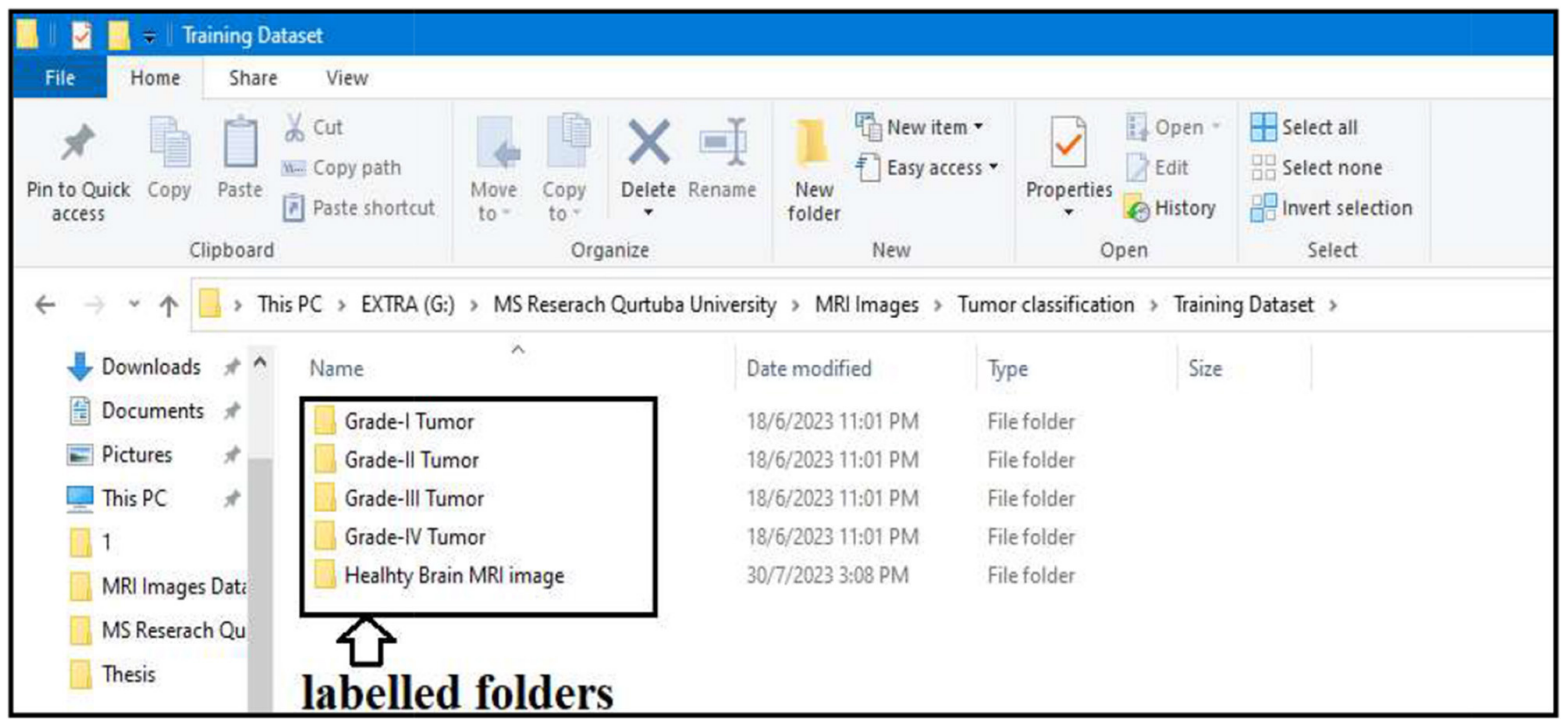
Showing labeled folders.

All the resultant images of step two are divided into two categories. One category is labeled images, and the second is unlabeled images. As we can see in Figure 16, the labeled images are used to train our segmentation model. The labeled images are stored in separate folders (Grade I Tumor, Grade II Tumor, Grade III Tumor, Grade IV Tumor, and Healthy Brain MRI image). The remaining unlabeled images will be used in the testing phase of the segmentation model. Approximately 90% of the images were labeled for training purposes, and the remaining 10% was unlabeled and allocated for testing the segmentation model.

#### 4.2.2 Loading all labeled images

**Figure 17:**
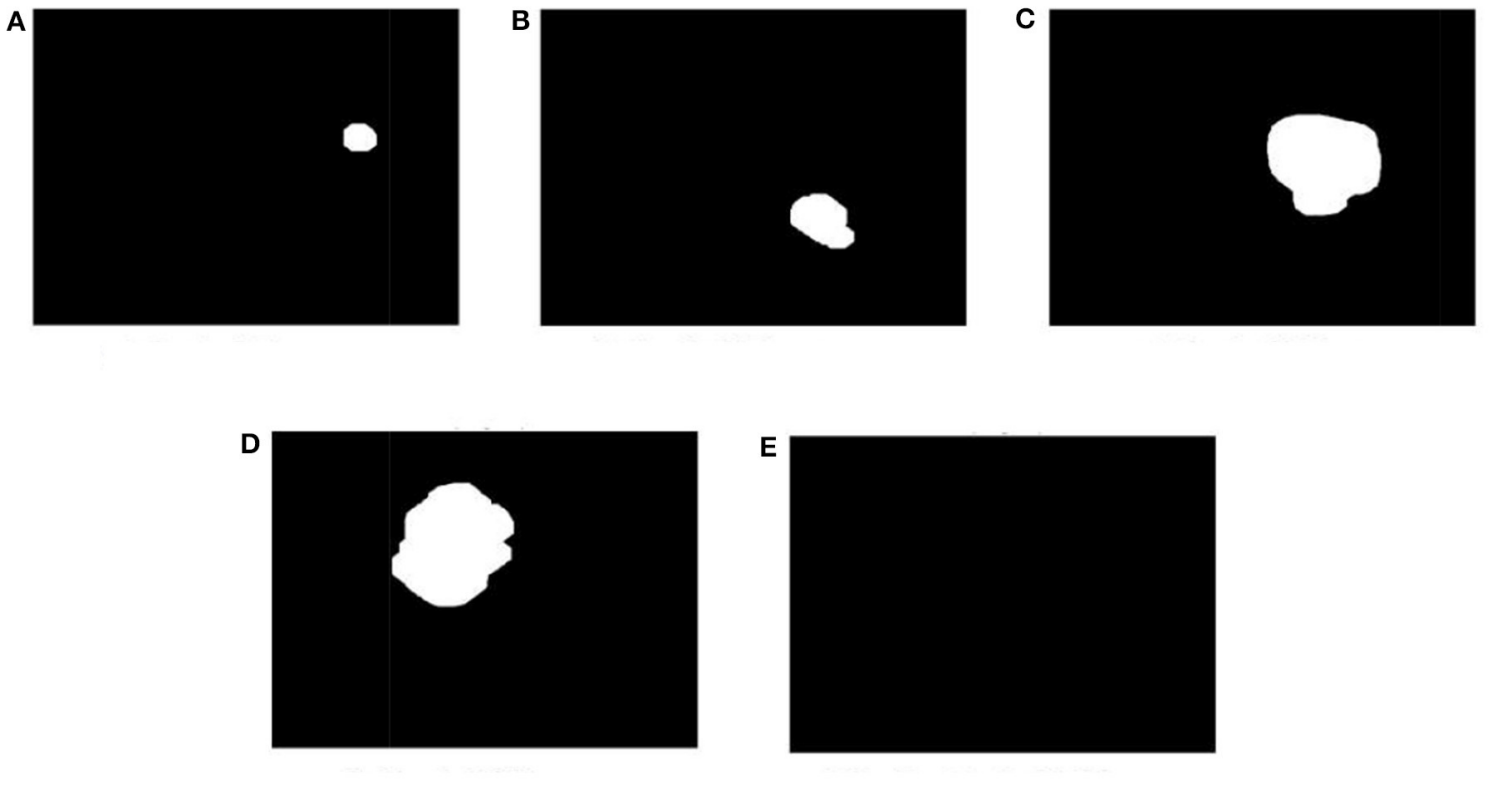
Display a few images from all the labeled folders. **(A)** Grade I tumor. **(B)** Grade II tumor. **(C)** Grade III tumor. **(D)** Grade IV tumor. **(E)** Healthy brain MRI image.

All images stored in the labeled folders are loaded into the segmentation model for training purposes. They are loaded at once and stored as a variable described in the algorithm implementation; the labeled images stored as a variable are further used to train our segmentation model. Figure 17 displays a few images from all the labeled folders stored in a variable.

#### 4.2.3 Development of the training phase

**Figure 18:**
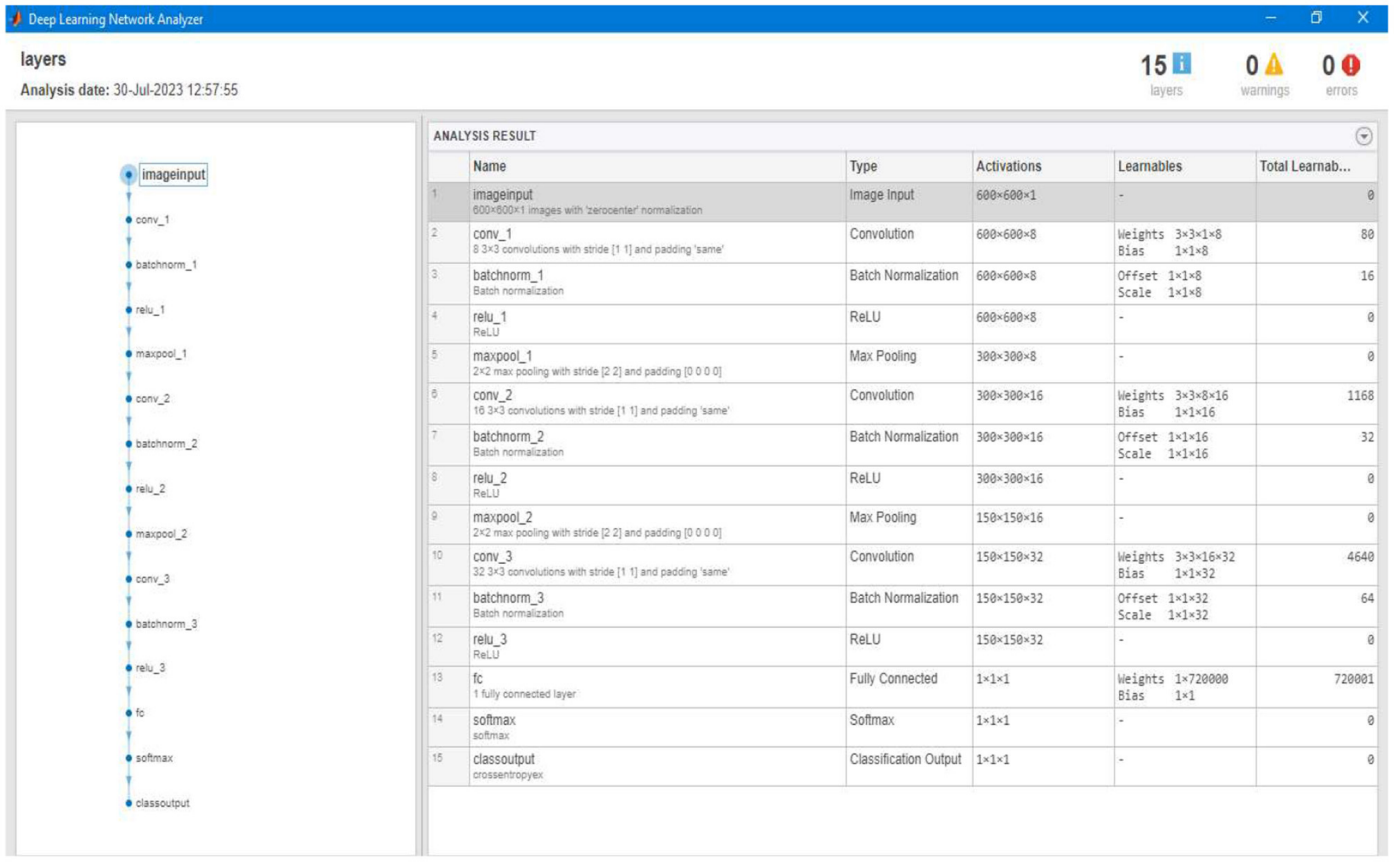
Showing different layers of the overall training model constructed to train the segmentation model.

After labeling and loading the data, the segmentation model will train itself based on the parameter/labeled images. The model trains based on the size of tumor types already set in the labeled data and stored as a variable in the previous step. Figure 18 shows the different layers of the training phase of our segmentation model. The training model consists of three convolution layers and one fully connected layer.

#### 4.2.4 Training the segmentation model

**Figure 19:**
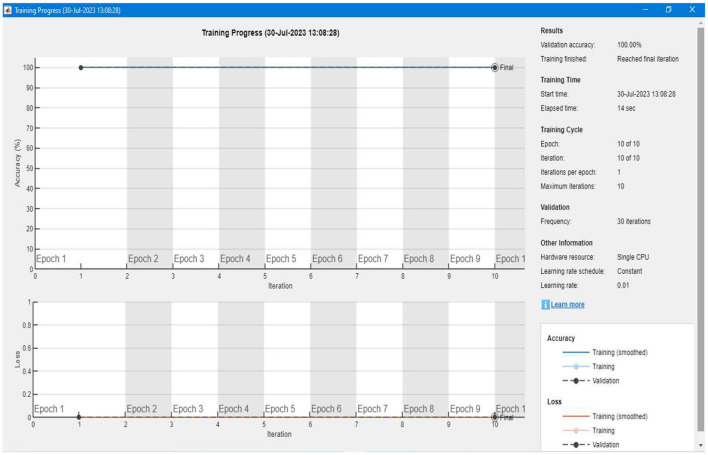
Showing accuracy and validation of the training phase.

After setting up the labeled data and developing the training model, the algorithm trains itself using the labeled data. Figure 19 shows a detailed representation of the training and validation of our proposed segmentation model. We can see that the accuracy of the training is 100%, and there is a 0% loss in the validation of the data. For each image, the model takes an average of 10 to 12 seconds of computation time to complete the overall training.

#### 4.2.5 Testing and segmentation results

**Figure 20:**
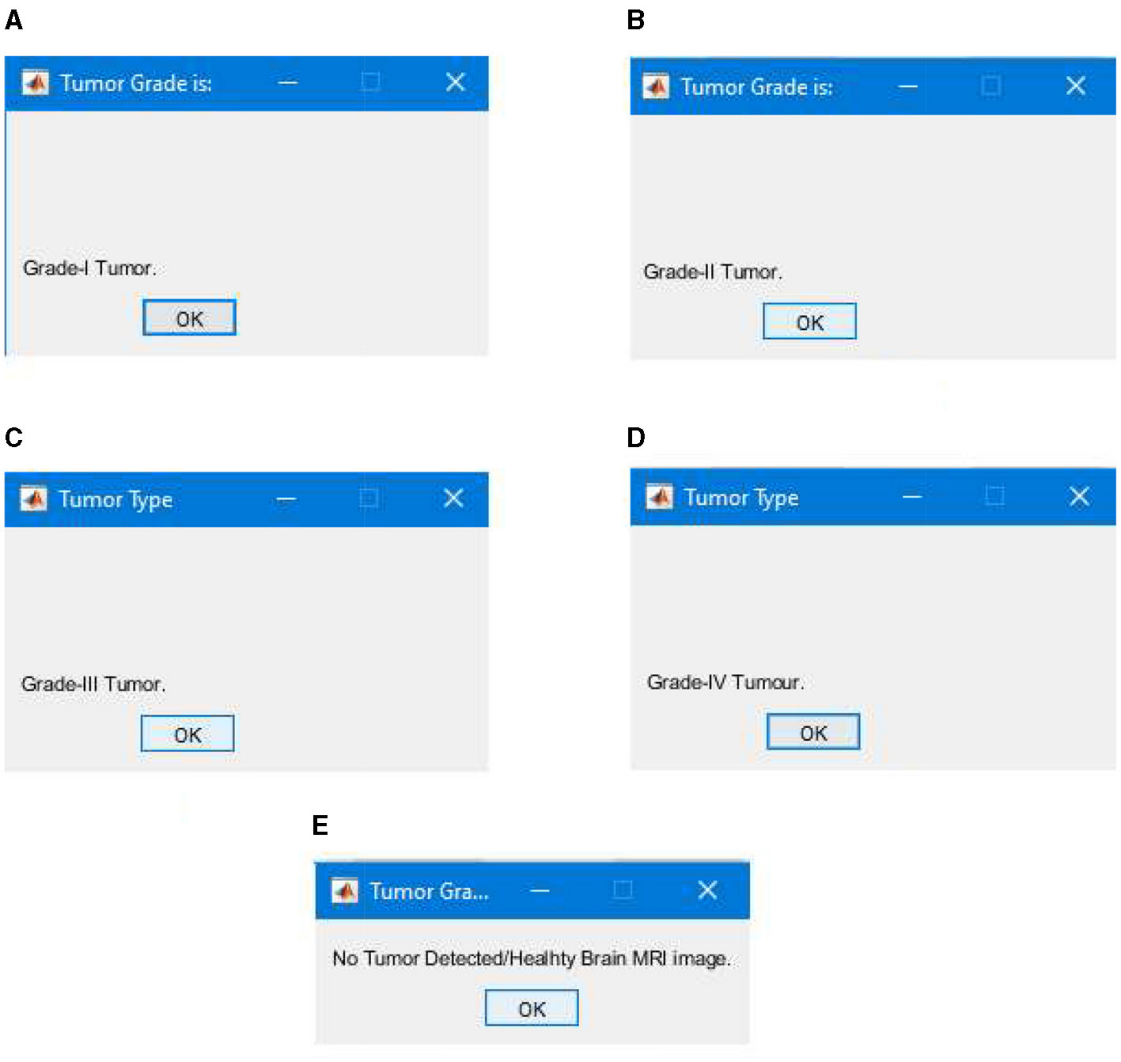
Segmentation results classifying tumor types into grades, **(A)** successful segmentation results of Grade I, **(B)** successful segmentation results of Grade II, **(C)** successful segmentation results of Grade III, **(D)** successful segmentation results of Grade IV, and **(E)** successful segmentation results of healthy brain MRI image with no brain tumor detected.

After completing the training, we tested our segmentation model; the testing was performed using the unlabeled images comprising 10% of the total images/data. The testing phase of our segmentation model achieved an overall true segmentation rate of 100%. All the tumor types were successfully classified into their respective grades (from Grade I to Grade IV). The model also accurately categorized the healthy brain MRI images, distinguishing them from those with tumors. The overall achievement of our proposed methodology showed significance and efficacy. Figure 20 illustrates a few results generated during the segmentation of the proposed segmentation model. We can see that the tumor regions in the brain MRI images are successfully classified into their respective grades, and the healthy brain MRI image is also successfully classified without detecting any tumor region.

### 4.3 Comparison of proposed and current research work

In this section, we compare our proposed model with existing research. The criteria for comparing are the overall accuracy of the segmentation model, precision, recall, and F-measure. In Table 2, the results of our proposed model are compared with existing research. Based on the comparison, it is evident that our proposed research model performs better than existing research models.

**Table 2:** Comparison of proposed and existing models.

**Method**	**No. of tumor types**	**Segmentation of tumor into grades**	**Segmentation results**			
			**Accuracy**	**Precision**	**Recall**	**F-Measure**
Convolutional neural networks (CNN) using grid search optimization (Irmak, 2021)	A total of five tumor types	Only Glioma brain tumors are classified into grades II, III, and IV	98.14%	98.31%	——	——
Multiclass support vector machine (M-SVM) (Maqsood et al., 2022)	Three tumor types	——	98.92%	——	——	——
Four models of deep convolutional neural networks: inceptionresnetv2, inceptionv3, transfer learning, brain-tumor-net model (Shoaib et al., 2022)	Three tumor types and normal cases	——	86.80%, 85.34%, 93.15%, 91.24%	86.85%, 85.12%, 93.14%, 91.20%	——	86.83%, 84.98%, 93.11%, 91.08%
Histogram differencing and KNN (Nida-Ur-Rehman et al., 2017)	Four tumor types and healthy brain MRI images	——	97.3%	——	——	——
Proposed Model: binary convolution neural network (BCNN)	Ten tumor types and healthy brain MRI images	Into four grades (Grade I to Grade IV) and healthy brain MRI images	99.40 %	99.32%	99.45%	99.28%

## 5 Conclusion and future work

In this study, we proposed a new model based on deep learning BCNN to classify the most common ten types of brain tumors into grades (from Grade I to Grade IV) based on the size and growth of the tumor. The tumor types that were considered in our research work are metastatic or secondary tumors, Meningioma, CNS Lymphoma, Glioblastoma, Astrocytoma, Pituitary Adenoma, Ependymomas, Medulloblastomas, Oligodendroglia's, and Hemangioblastomas. A dataset of 6,600 MRI images was used, including all types of tumor MRI images and healthy brain MRI images. The dataset was collected from the two main sources (Nida-Ur-Rehman et al., 2017; Radiopaedia's, 2023) and verified by an FCPS neurosurgeon for their validity.

The methodology that we proposed had two phases. The first phase consisted of preprocessing and conversion to binary and morphological operations. The image was loaded in step one, and its intensity and contrast were set to get more accurate results in the next step and phase two. We used the two-dimensional adaptive filter to filter and set up the image contrast for this. Next, during preprocessing, we standardized the size of all the MRI images to 600^*^600 using the nearest neighbor interpolation and converted all the images to a standard size. In step two of phase one, all the images were converted into binary. Morphological operations were performed to remove any noise or unwanted pixels that could result in errors or give false segmentation results.

In step two, we executed the main segmentation model based on the BCNN developed using three convolution layers and one fully connected layer. The images that were generated after step two of phase one were divided into two categories: labeled and unlabeled. The labeled images were stored in folders named according to tumor grades (Grade I to Grade IV) and healthy brain MRI images. The labeled images were used to train our algorithm; overall, 90% of the images were labeled and used to train our algorithm. In the next step, we tested our algorithm using the remaining 10% of images that were unlabeled.

The overall results of our segmentation model were very satisfactory, with 99.40% accuracy, 99.32% precision, 99.45% recall, and 99.28% F-Measure score. These results demonstrate the significance and efficacy of our proposed model, which successfully classified all the tumor types into their respective grades (from Grade I to Grade IV). Our study has also curated a new dataset for the research community with more than 6,000 brain MRI images that contain the 10 most common types of brain tumor and healthy brain MRI images. This robust framework enhances the accuracy of brain tumor segmentation and sets a new benchmark for early detection and grading of brain tumors, thereby contributing to the advancement of neuro-oncological diagnostics. The technique provides a more effective contribution to the clinical practitioner to easily, quickly, and accurately classify and segment brain tumors in the early stages, which will help them provide better treatment to patients suffering from this deadly disease. This model can effectively reduce the number of deaths caused by brain tumors by facilitating early and accurate detection of brain tumors and treatment.

### 5.1 Future work

Going forward, this study will aim to address the following challenges and limitations of the proposed research work and methodology:

- There are more than 100 types of brain tumors; these can be included for segmentation into grades.
- Other imaging technologies, such as CT scans, can be used along with MRI images to classify the brain using different deep learning techniques.
- Different deep-learning techniques can be used to classify other tumor types effectively using RGB images.

## Data availability statement

The datasets presented in this study can be found in online repositories. The names of the repository/repositories and accession number(s) can be found in the article/supplementary material.

## Author contributions

AZ: Conceptualization, Supervision, Writing – review & editing. LK: Conceptualization, Formal analysis, Methodology, Writing – original draft, Writing – review & editing. QR: Formal analysis, Methodology, Writing – original draft. TR: Conceptualization, Data curation, Writing – review & editing. MS: Conceptualization, Validation, Writing – review & editing. SA: Funding acquisition, Visualization, Writing – review & editing. MI: Conceptualization, Formal analysis, Writing – review & editing. MN: Resources, Validation, Visualization, Writing – review & editing. SH: Conceptualization, Formal analysis, Writing – review & editing. HM: Data curation, Formal analysis, Funding acquisition, Writing – review & editing.
