# Corrigendum: An enhanced pattern detection and segmentation of brain tumors in MRI images using deep learning technique

**DOI:** 10.3389/fncom.2025.1570979

**Published:** 2025-06-03

**Authors:** Lubna Kiran, Asim Zeb, Qazi Nida Ur Rehman, Taj Rahman, Muhammad Shehzad Khan, Shafiq Ahmad, Muhammad Irfan, Muhammad Naeem, Shamsul Huda, Haitham Mahmoud

**Affiliations:** ^1^Qurtuba University of Science and Information Technology, Peshawar, Pakistan; ^2^Abbottabad University of Science and Technology, Abbottabad, Pakistan; ^3^Institute of Management Sciences (IMSciences), Peshawar, Pakistan; ^4^Department of Industrial Engineering, College of Engineering, King Saud University, Riyadh, Saudi Arabia; ^5^Department of Computer Science, Kohat University of Science and Technology, Kohat, Pakistan; ^6^School of Information Technology, Deakin University, Burwood, VIC, Australia

**Keywords:** brain tumor, deep learning, pattern detection, neuroscience, segmentation technique, convolution neural network, binary convolution neural network, magnetic resonance images

In the published article, in the Section **3. Materials and methods**, *3.1 Dataset of brain MRI images*, 10 references and citations were erroneously omitted from the subheadings “CNS Lymphoma,” “Glioblastoma,” “Meningioma,” “Metastases,” “Astrocytoma,” “Pituitary Adenoma,” “Ependymomas,” “Medulloblastomas,” “Oligodendrogliomas,” and “Hemangioblastomas”. The corrected paragraphs with citations included, and a corrected Reference list, appears below:

“**CNS Lymphoma**: Primary central nervous system Lymphoma is a type of brain tumor that can be primary and secondary; in this type of tumor, cells emerge in the lymphoma and/or the spinal cord region (RMH Neuropathology, 2013).

**Glioblastoma**: This type of tumor is considered dangerous because it grows fast and spreads quickly inside the brain. Initially, glioblastoma attacks adjacent brain tissues (Gaillard, 2018).

**Meningioma**: This brain tumor starts inside the brain tissues called meninges that protect the brain and spinal cord. Most meningiomas are not dangerous but can reach up to Grade III tumor levels (Di Muzio, 2023).

**Metastases**: These types of tumors spread from other parts of the body, such as the lungs, breasts, and kidneys, to the rest of the body. Once they spread to the brain, they can create one or more tumors inside the brain (Brusic, 2021).

**Astrocytoma**: These tumors can be cancerous or non-cancerous. Some grow very slowly, while others can be aggressive. They appear first in cells called astrocytes (Gaillard, 2021).

**Cystic Pituitary Adenoma and Meningioma**: They are generally slow-growing types and fall in the benign category of brain tumors, which are mostly considered Grade 1 or Grade II tumors. Most patients with this type of tumor are diagnosed after several years before observing any signs. It develops from pituitary tissues and grows inside the pituitary gland of the brain (Gaillard, 2016).

**Ependymomas**: This type of brain tumor develops inside the brain or spinal cord area. It can reach Grade 3 from Grade 1. It initially begins in ependymal cells that help to maintain and improve brain streams (Schubert, 2011).

**CNS Embryonal Tumor NOS**: It is the most common type of brain tumor found in children <3 years of age. By nature, this type of tumor is malignant, and it exists in the area of the cerebellum as a solid mass (Jones, 2021).

**Oligodendrogliomas**: They emerge around the brain and cortex, the brighter white portion of the brain. They are most commonly considered the middle-aged adult's tumor (Gaillard, 2010).

**Hemangioblastomas**: They are benign brain tumors that mostly rise around the brain, spinal cord, and behind the eye tissues (retina). They normally occur in young and middle-aged people. (Balachandran, 2010).

In the published article, in Section **3. Materials and methods**, *3.1 Dataset of brain MRI images*, the title of the paragraphs “Pituitary Adenoma” and “Medulloblastomas” were incorrectly mistyped. The correct titles of the paragraphs should have been “Cystic Pituitary Adenoma and Meningioma” and “CNS Embryonal Tumor NOS” respectively.

In the published article, there was an error in Figure 1 as published, where “Pituitary Adenoma” and “Medulloblastomas” were mislabeled. This should have been “Cystic Pituitary Adenoma and Meningioma” and “CNS Embryonal Tumor NOS” respectively.

The corrected Figure 1 and its caption appear below:

**Figure 1:**
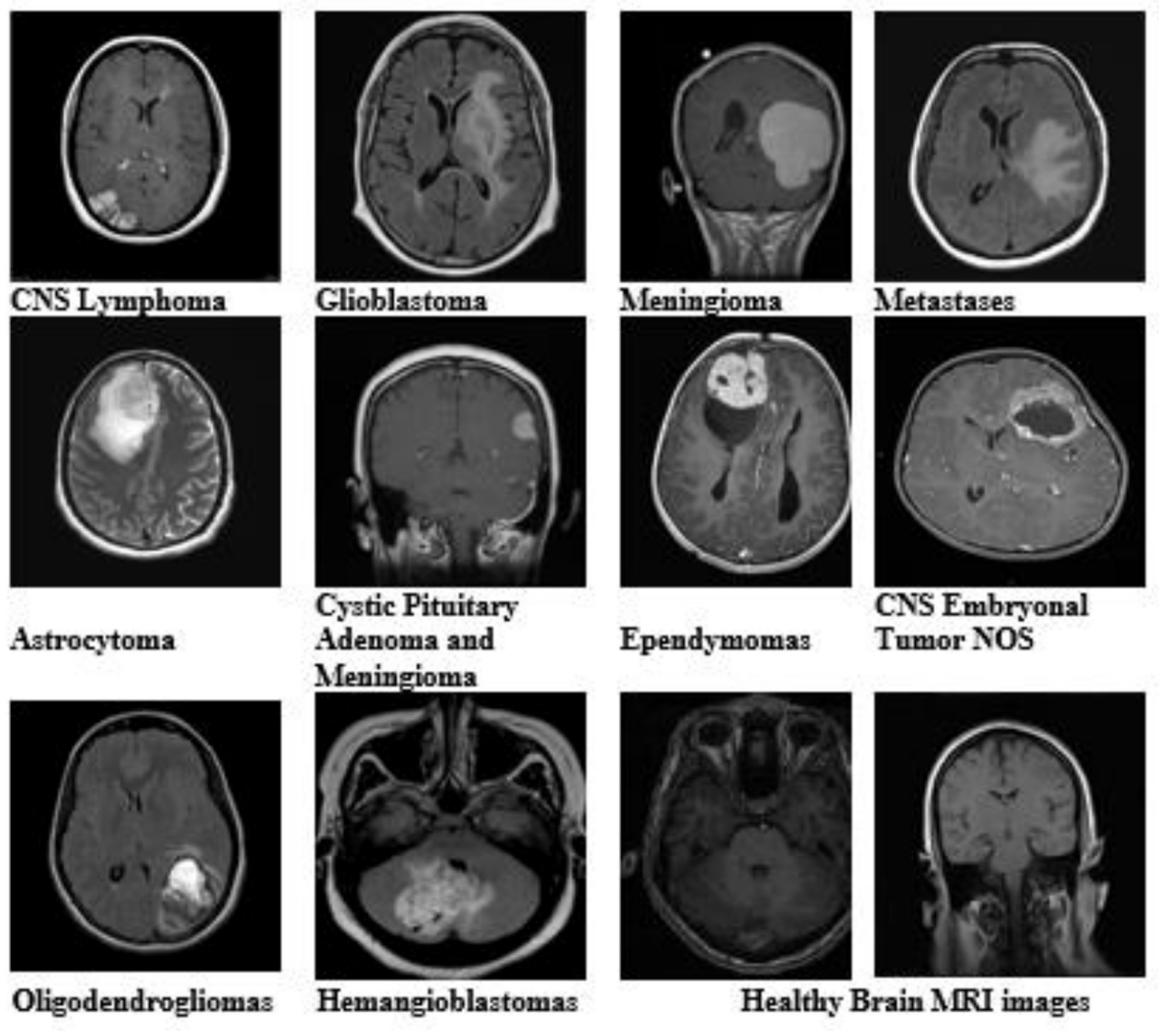
Brain MRI images of all tumor types and healthy brain MRI images from the dataset with different variants and modalities.

In the published article, there was an error in Table 1 as published, where in the column “Tumor Type” “Pituitary Adenoma” and “Medulloblastomas” were mislabeled. These should have been “Cystic Pituitary Adenoma and Meningioma” and “CNS Embryonal Tumor NOS” respectively.

The corrected Table 1 and its caption appear below:

**Table 1:** Dataset of brain MRI images of each tumor type and healthy brain MRI images.

**S. No**	**Tumor types**	**Number of MRI images**
1	CNS Lymphoma	800
2	Glioblastoma	600
3	Meningioma	600
4	Metastases	400
5	Astrocytoma	600
6	Cystic Pituitary Adenoma and Meningioma	500
7	Ependymomas	600
8	CNS Embryonal Tumor NOS	500
9	Oligodendrogliomas	500
10	Hemangioblastomas	500
11	Healthy brain MRI images	1,000
Total		6,600

The authors apologize for these errors and state that they do not change the scientific conclusions of the article in any way. The original article has been updated.
